# A Curious Case of Biopsy-Proven Usual Interstitial Pneumonia

**DOI:** 10.7759/cureus.47661

**Published:** 2023-10-25

**Authors:** Austin Makadia, Vishesh Persaud, Asad Chohan, Saiara Coudhury, Abhay Vakil

**Affiliations:** 1 Pulmonary Medicine, Corpus Christi Medical Center, Corpus Christi, USA

**Keywords:** idiopathic pulmonary fibrosis (ipf), usual interstitial pneumonia (uip), indeterminate uip, idiopathic pulmonary fibrosis, interstitial lung disease, usual interstitial pneumonia

## Abstract

Usual interstitial pneumonia (UIP) refers to a combination of radiologic and histologic findings, which include patchy interstitial fibrosis with fibroblastic foci and dense acellular collagen that causes architectural distortion due to scarring and honeycomb change with alternating areas of normal lungs. The UIP pattern is not a synonymous term with idiopathic pulmonary fibrosis (IPF). IPF is diagnosed when an etiologic workup has been performed, deemed to be unrevealing, with a radiologic or histologic UIP pattern. While the 2018 American Thoracic Society (ATS)/European Respiratory Society (ERS) guideline categories of UIP help eliminate the need for surgical lung biopsy (SLB) in two categories, i.e., “definite UIP” and “probable UIP,” when characterizing a patient in the other categories, clinicians should wary about prolonging SLB in patients to determine the fibrosis pattern. Changes in the treatment and overall prognosis of patients can occur due to SLB confirming a UIP pattern on histology. Here, we report the case of a patient with an indeterminate UIP pattern on high-resolution computed tomography (HRCT) with histopathologic diagnosis of UIP on SLB. With no underlying identifiable cause for the UIP pattern, the patient was diagnosed and managed as IPF, ultimately requiring lung transplantation. This case highlights the importance of pursuing surgical lung biopsy in patients with indeterminate UIP on HRCT scanning to facilitate prompt treatment and guide further management.

## Introduction

Usual interstitial pneumonia (UIP) refers to a combination of radiologic and histologic findings, which include patchy interstitial fibrosis with alternating area of a normal lung, temporal heterogeneity of fibrosis characterized by scattered fibroblastic foci with dense acellular collagen, and architectural distortion due to chronic scarring or honeycomb change [1]. Idiopathic pulmonary fibrosis (IPF) and UIP are terms that are often used interchangeably. However, UIP is associated with other conditions, including but not limited to connective tissue disorders, hypersensitivity pneumonitis, familial interstitial lung disease (ILD), and heavy metal exposure. Therefore, diagnosing UIP in a patient is not the same as diagnosing IPF, which requires exclusion of the said underlying clinical conditions and associations. 

IPF is a lung parenchymal disease in which no known underlying etiology is found, with a UIP pattern found on radiology or on surgical lung biopsy (SLB) histopathology. SLB is an established method of obtaining adequate samples for histopathological examination (HPE). If no reliable HPE is obtained, a compatible composite of clinical and radiologic findings must correlate in order to label the disease process as IPF [1]. The exact role of imaging studies and SLB in patients with a UIP pattern in establishing the diagnosis of IPF has been reconsidered over the last decade. High-resolution computed tomography (HRCT) has notably improved the diagnostic yield for both UIP and IPF. According to the American Thoracic Society (ATS)/European Respiratory Society (ERS) guidelines for IPF, HRCT findings have been divided into four distinct diagnostic categories for the diagnosis of a UIP pattern, which include 1) definite UIP pattern, 2) probable UIP pattern, 3) indeterminate for UIP pattern, and 4) alternative diagnosis, depending on the presence or absence of classic features of UIP [2]. The presence and extent of radiologic findings, such as ground-glass opacities, cystic changes, mosaic attenuation, micronodule formation, consolidation, pleural plaques, lymphadenopathy, and distribution, including peribronchovascular, perilymphatic, upper or middle lung predominance, and associated pleural effusion/thickening, determine how the HRCT is categorized. 

A diagnosis of UIP can be made with a great degree of accuracy in patients with typical radiologic findings resulting in a less frequent pursuit of SLB for these patients. For patients with findings of categories 3 and 4, the SLB with HPE remains a critical step to obtain a diagnosis of UIP and is the single most important predictor of prognosis at the time of discovery; therefore, it continues to be the gold standard for diagnosis [3]. Here, we report the case of a patient with an indeterminate UIP pattern on HRCT with histopathologic diagnosis of UIP on SLB. With no underlying identifiable cause for the UIP pattern, the patient was diagnosed and managed as IPF, ultimately requiring lung transplantation. This case highlights the importance of pursuing surgical lung biopsy in patients with indeterminate UIP on HRCT imaging to facilitate prompt treatment and guide further management.

## Case presentation

A 65-year-old-male patient presented to the clinic for evaluation of chronic cough that had been ongoing for six months. Cough was non-productive, intermittent, without any aggravating or relieving factors. The patient had intermittent episodes of dry cough during the day and night. There was no specific relation of cough with any specific exposures or with eating. He was treated twice with oral steroids and azithromycin over the course of six months without any symptomatic relief for possible pneumonia or asthma exacerbations. The patient denied any associated post-nasal drip, heartburn, weight loss, or hemoptysis. He denied fever, chills, night sweats, sick contacts, or any recent travel history. The patient did complain of associated shortness of breath that had been progressively worsening and was unable to walk to his mailbox without stopping to catch his breath. He denied any significant environmental or occupational exposures and was up-to-date with immunizations, including pneumococcal, flu, and initial COVID-19 shots. The patient did not have any birds or pets at home. He was a lifetime non-smoker, without any alcohol or drug use, and without any medical problems. The patient denied any family history of pulmonary disorders. 

On examination, the patient had bilateral basilar crackles. He had no oropharyngeal exudation, clubbing, extremity edema, or cyanosis. Cardiac examination was normal. Ocular exam did not reveal erythema, irritation, blurring, and watering, and vision was intact. Skin exam revealed no rashes, erythema, or scratch marks. Joint exam was unremarkable as well, without any erythema, swelling, stiffness, or crepitus, and exhibited a normal full range of motion.

**Figure 1:**
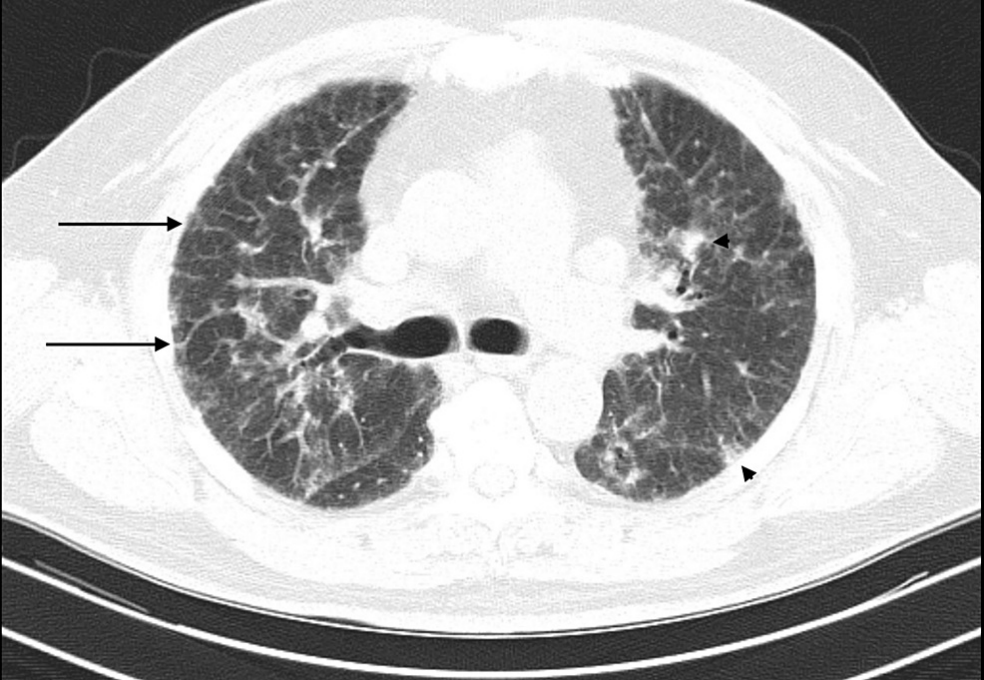
High-resolution computed tomography showing interstitial thickening with reticulation and ground-glass opacities Long arrows: reticulation; arrow heads: areas of ground-glass opacities

**Figure 2:**
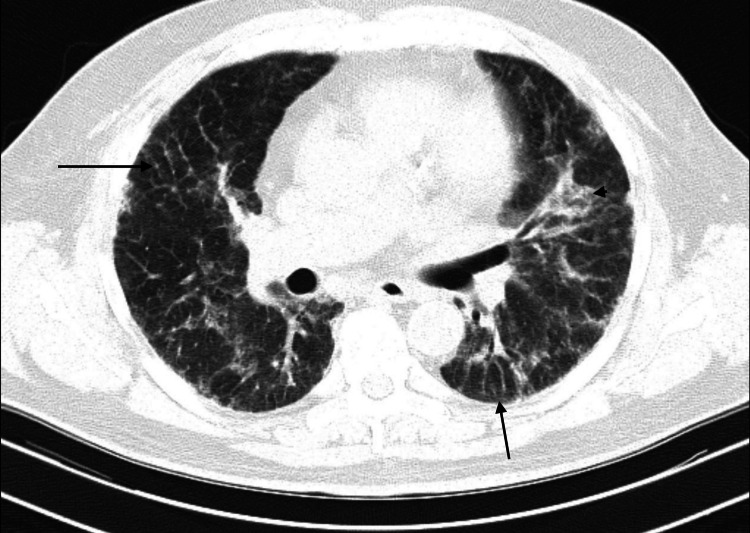
High-resolution computed tomography further down the thorax, illustrating further interstitial thickening with reticulation and ground-glass opacities Long arrow: reticulation; arrow head: areas of ground-glass opacities

**Figure 3:**
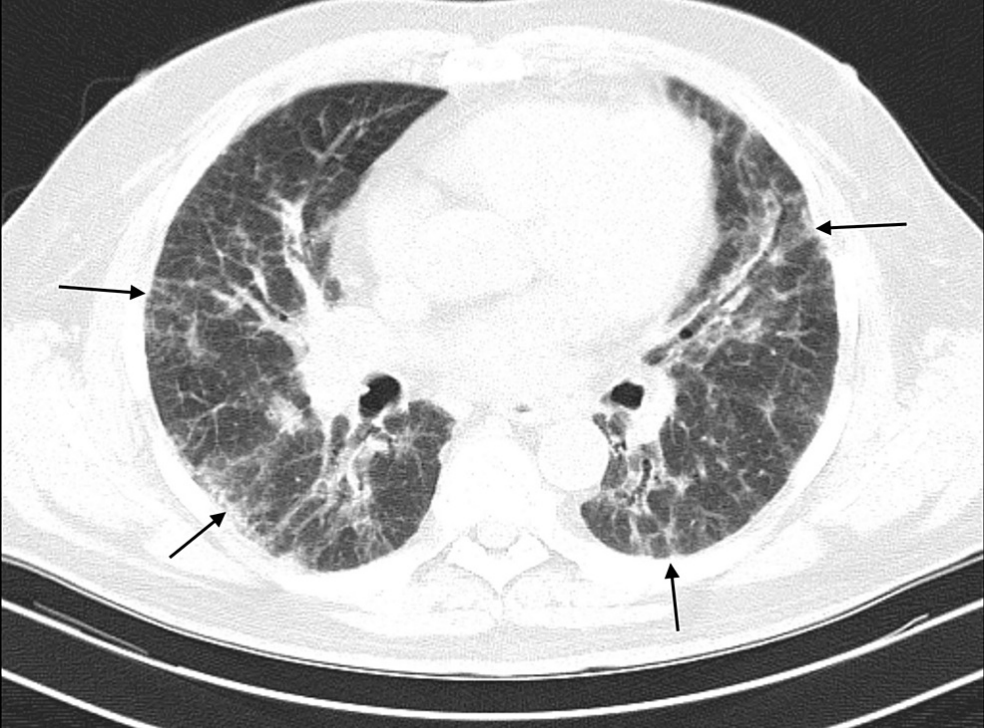
High-resolution computed tomography images illustrating further interstitial thickening with subpleural reticulation Long arrow: reticulation

**Figure 4:**
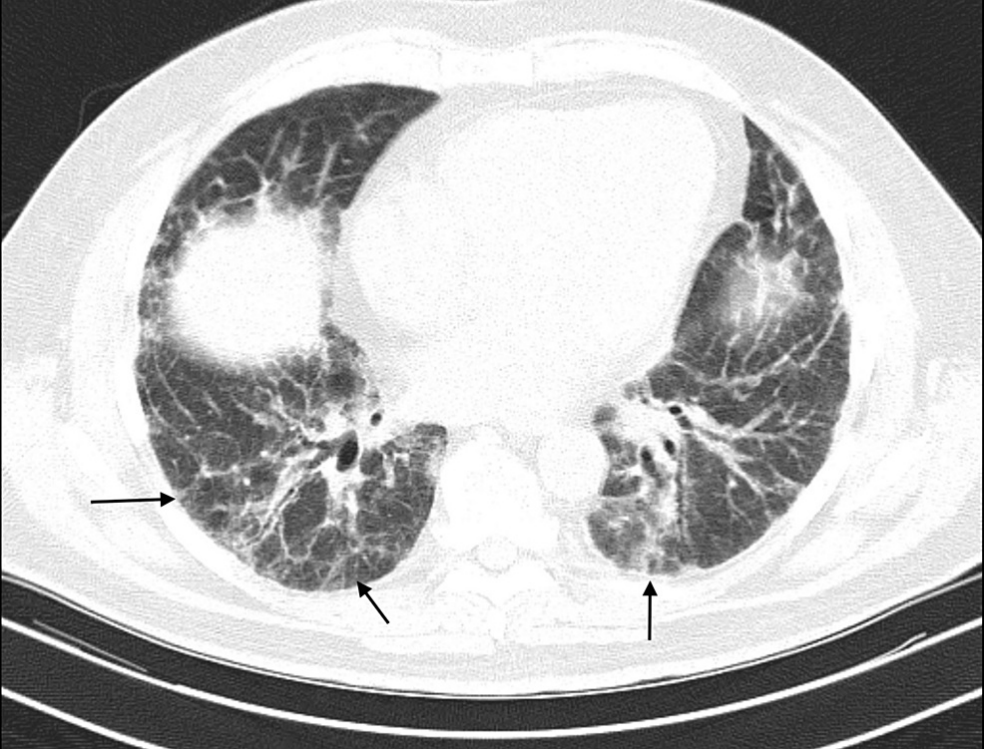
High-resolution computed tomography of lung bases showing subpleural reticulation more prominent posteriorly. Long arrow: reticulation

Pulmonary function testing was consistent with severe restriction with a forced vital capacity (FVC) of 1.42 L, 45% predicted. HRCT showed bilateral interlobular and intralobular interstitial thickening, subtle reticulation with areas of ground-glass opacities without any associated bronchiectasis or honeycombing (Figures 1-4). These findings were consistent with the indeterminate UIP pattern. A six-minute-walk test showed severe activity limitation of 120 feet, and the patient desaturated with ambulation to 65% requiring home oxygen therapy.

The patient had a bronchoscopy to evaluate for underlying infections. Immunologic and rheumatologic workup was sent. All test results, including anaerobic and aerobic cultures, fungal cultures, acid-fast-bacilli culture, cytology, and CD4 and CD8 cell counts, were unrevealing. Laboratory testing, including creatine kinase, ribonucleic acid polymerase III, anti-Smith antibodies, ribonucleoprotein antibodies, rheumatoid factor and cyclic citrullinated peptide, myeloperoxidase antibody, proteinase-three antibody, smooth muscle antibodies, Sjogren’s antibodies, angiotensin-I-converting enzyme level, myositis panel, antinuclear antibody screen, and double-stranded deoxyribonucleic-acid antibody, was negative. 

**Figure 5:**
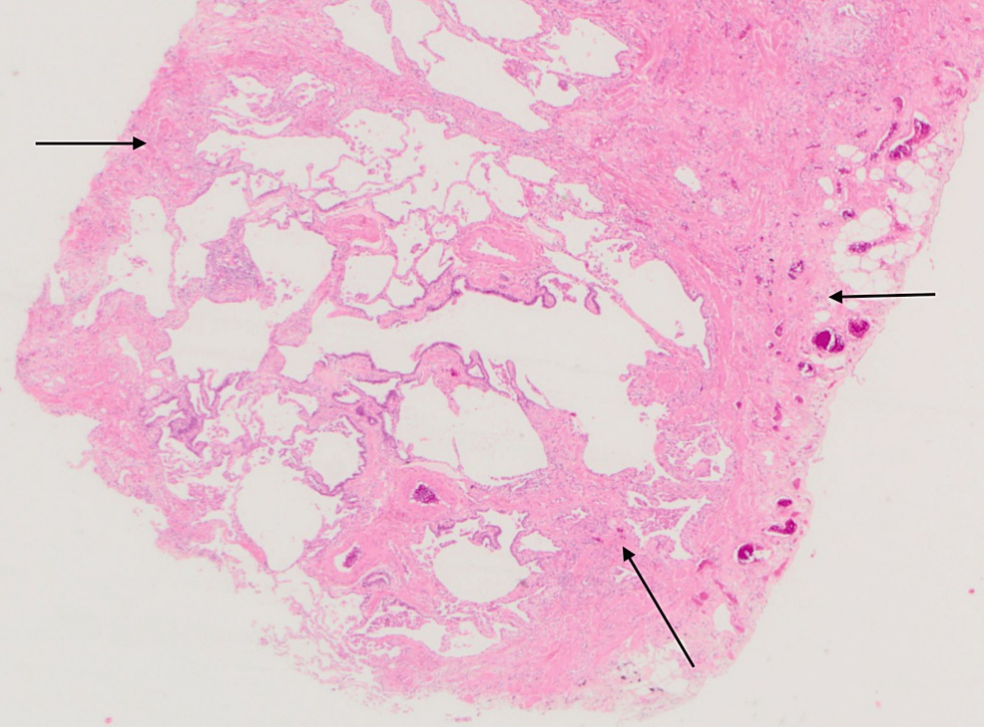
Histology slide illustrating peripheral dense fibrosis adjacent to normal parenchyma on low-power magnification, along with some honeycombing in the center Long arrows: areas of fibrosis

**Figure 6:**
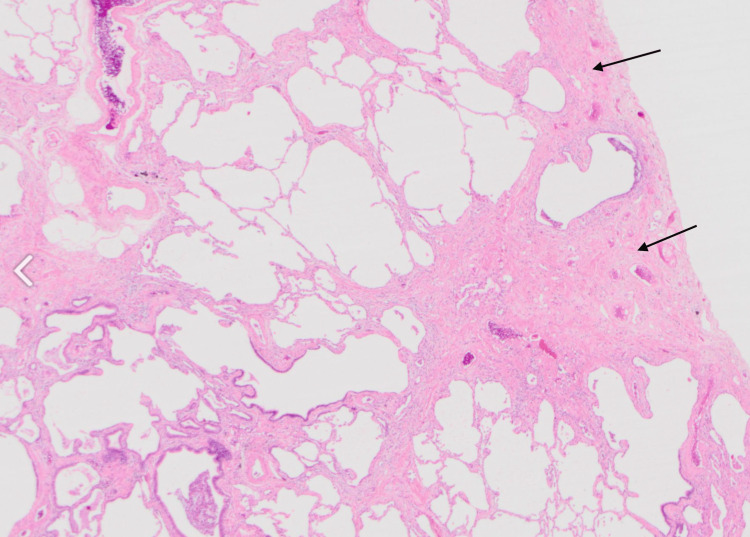
Histology slide illustrating peripheral dense fibrosis adjacent to normal parenchyma, along with some honeycombing in the center Long arrows: areas of fibrosis

**Figure 7:**
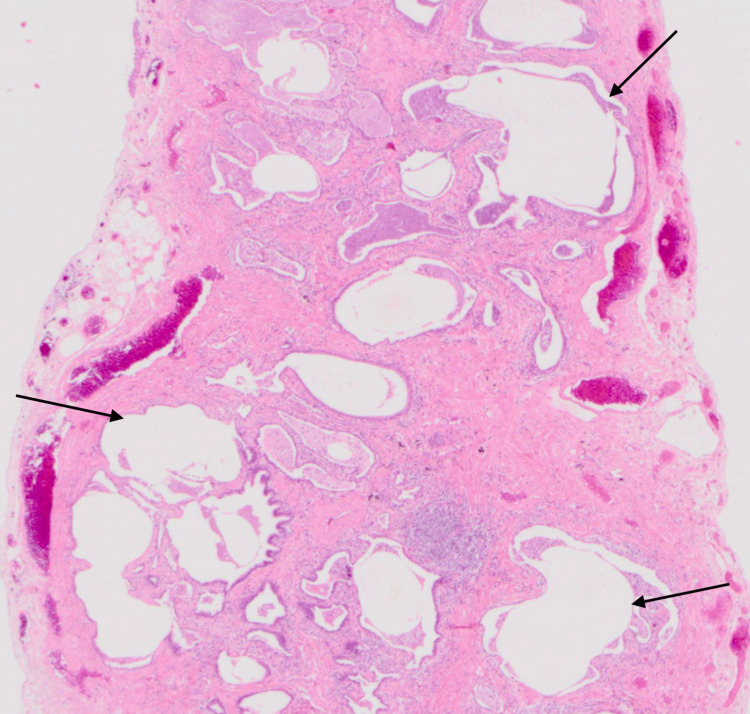
Histology slide illustrating honeycombing, with destruction of normal alveoli histology Long arrows: honeycombing

**Figure 8:**
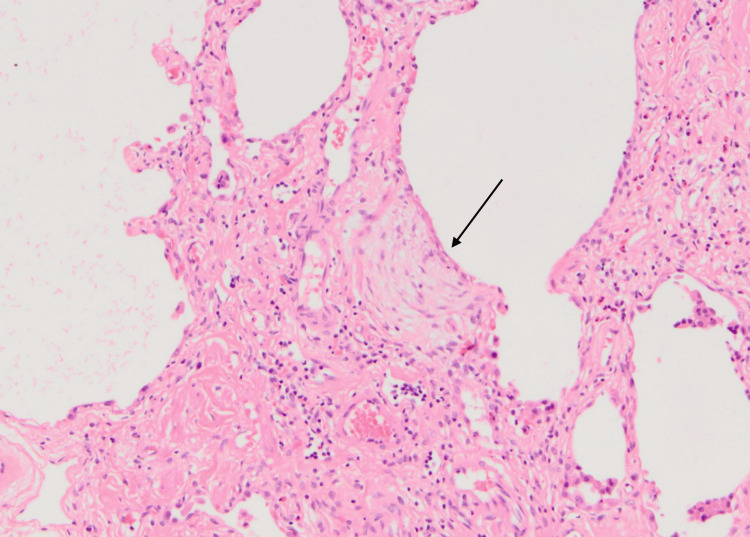
Histology slide illustrating a fibroblastic focus, which is typically found in usual interstitial pneumonia Long arrow: fibroblastic focus

**Figure 9:**
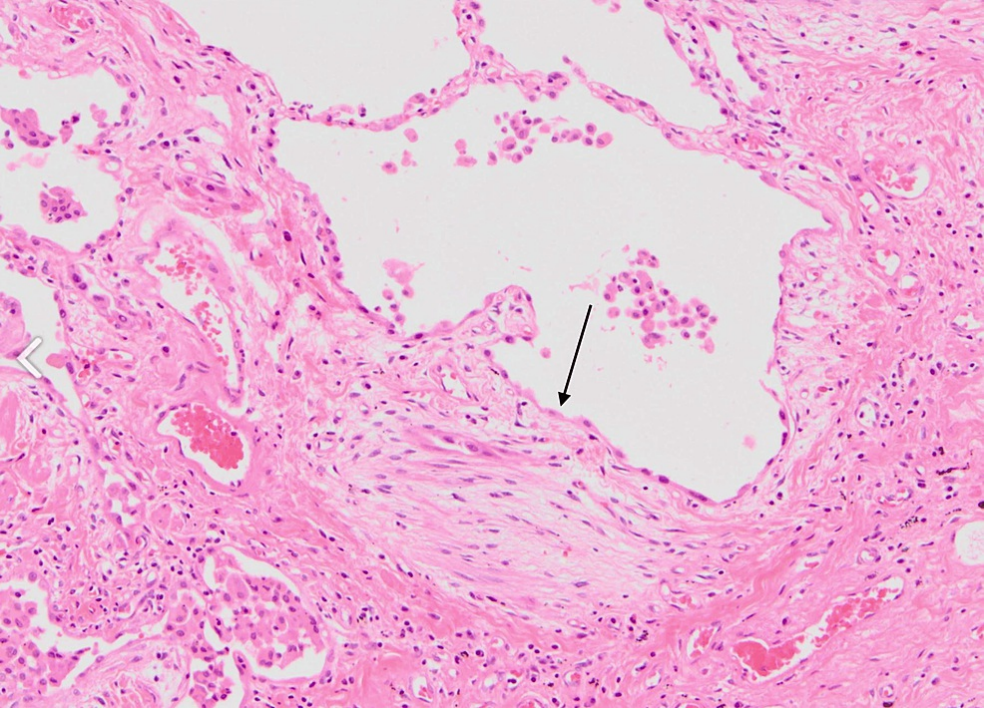
Histology slide illustrating another example of fibroblastic focus Long arrow: fibroblastic focus

**Figure 10:**
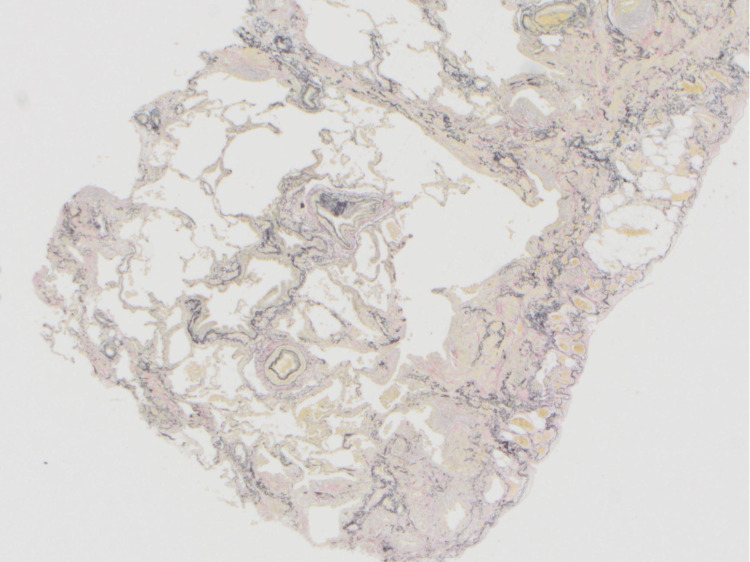
Pathology slide illustrating staining with an Elastica-van Gieson staining x2 to highlight fibrosis.

An SLB was performed through a video-assisted thoracoscopic approach. Multiple samples were collected from the right upper, middle, and lower lobes. Histopathologic examination showed a widespread extensive destruction of the normal lung parenchyma, with largely subpleural old dense fibrosis containing numerous honeycombing areas, and fibroblastic foci, without granulomas or necrosis (Figures 5-10). These findings were consistent with UIP. In the absence of any identifiable cause, the patient was diagnosed as having IPF and started on pirfenidone therapy and was referred for lung transplantation. The patient did not show any significant clinical difference and the lung function stabilized without significant deterioration in the next three months while being on pirfenidone. He underwent bilateral lung transplantation after three months of drug therapy followed by an uncomplicated post-transplant course, with improvement in his symptomatology and functional status.

## Discussion

Establishing whether a patient has lung parenchymal disease with a UIP pattern is very important for overall patient prognosis and management of the disease process. A UIP pattern with an underlying etiology and other fibrosis patterns have a different treatment approach and presumably more favorable prognosis than IPF with a possible reversibility of radiologic findings. Treatment approaches would consist of a treatment of the underlying disease process causing the fibrosis. If the UIP pattern was established and an underlying etiology was not found, patients would be diagnosed as having IPF, and a treatment with antifibrotics, symptomatic management, pulmonary rehabilitation, and early referral to transplantation center are key. The diagnosis of IPF comes with a poorer prognosis for patients compared to other fibrosis patterns.

 In the past, patients with appropriate clinical and radiology findings and without underlying etiologies were referred for SLB to establish the diagnosis of UIP. If the workup for the underlying etiology was proven negative, the patient would be labeled as having IPF. The 2011 ATS guidelines may have reduced the reliance on SLB by introducing the four different categorizations of radiologic findings on HRCT, reducing the need of SLB in patients with a definite and probable UIP pattern on HRCT [4]. Patients who are found to have an indeterminate UIP pattern or an alternative diagnosis based on HRCT findings should promptly be scheduled for SLB to establish histologic diagnosis as highlighted by this case and by previously published studies [2]. If multiple disciplinary team rounds are available, discussion of the case and possible alternate procedures to obtain samples prior to SLB should take place. 

According to a study performed by Brownell et al., patients who had HRCT inconsistent with UIP had definite/probable UIP on biopsy in 22.7% of cases [5]. The SLB changed diagnoses to UIP patterns, which would lead to changes in their treatment options. One study by Tomassetti et al. investigated how SLB changed diagnostic confidence and changed therapeutic strategies in one-third of cases determined to be IPF or non-IPF fibrotic ILDs [6].

The most common radiological features found in patients with the inconsistent UIP radiology category were peribronchovascular distribution (44%), ground-glass opacities (39%), mosaic perfusion/air trapping (23%), and upper-mid lung predominance (16%) [5]. Even when clinical and radiographic data were added together to multivariate prediction models for patients with the “inconsistent UIP” HRCT model, it did not provide enough predictive value to rule in histopathological UIP for any combination of these features with a maximum positive predictive value (PPV) of only 38% [6]. Therefore, the ultimate gold standard still remains obtaining an SLB in order to secure a definite diagnosis of a UIP pattern or other ILDs in the absence of HRCT findings of definite/probable UIP or other ILD patterns. In our case, the radiologic findings were not consistent with definitive UIP. However, the histopathology did establish a definitive diagnosis changing our management to the initiation of antifibrotics and leading to early referral for lung transplantation.

## Conclusions

The UIP pattern is not a term synonymous with IPF. IPF can only be diagnosed when an etiologic workup has been performed, deemed to be unrevealing, and is associated with a radiologic or histologic UIP pattern. While the new 2018 guideline categories of UIP help eliminate the need for SLB in two categories, i.e., “definite UIP” and “probable UIP,” when characterizing HRCT in categories 3 and 4, i.e., “indeterminate UIP” and “alternative diagnosis,” clinicians should be cautious about delaying SLB to determine the fibrosis pattern. This case report highlights the importance of SLB in cases where HRCT scan is in category 3 (indeterminate UIP pattern) in obtaining the diagnosis and initiating the correct treatment for patients with IPF. Changes in the treatment and overall prognosis of patients can occur due to SLB confirming a UIP pattern on histology. In medical centers where multidisciplinary team rounds can occur, these cases should be discussed for a potential need of SLB with possible alternate tissue sampling procedures available. Patients could potentially benefit from disease-specific therapies in such cases.
